# Hypoxia in Human Obesity: New Insights from Inflammation towards Insulin Resistance—A Narrative Review

**DOI:** 10.3390/ijms25189802

**Published:** 2024-09-11

**Authors:** Maria Mirabelli, Roberta Misiti, Luciana Sicilia, Francesco S. Brunetti, Eusebio Chiefari, Antonio Brunetti, Daniela P. Foti

**Affiliations:** 1 Department of Health Sciences, University “Magna Græcia” of Catanzaro, 88100 Catanzaro, Italy; maria.mirabelli@unicz.it (M.M.);; 2 Operative Unit of Endocrinology, “Renato Dulbecco” University Hospital, 88100 Catanzaro, Italy; 3 Department of Experimental and Clinical Medicine, University “Magna Græcia” of Catanzaro, 88100 Catanzaro, Italy; roberta.misiti@studenti.unicz.it; 4 Operative Unit of Clinical Pathology, “Renato Dulbecco” Hospital, 88100 Catanzaro, Italy

**Keywords:** insulin resistance, inflammation, hypoxia, adipose tissue, micro-RNA, adipokine

## Abstract

Insulin resistance (IR), marked by reduced cellular responsiveness to insulin, and obesity, defined by the excessive accumulation of adipose tissue, are two intertwined conditions that significantly contribute to the global burden of cardiometabolic diseases. Adipose tissue, beyond merely storing triglycerides, acts as an active producer of biomolecules. In obesity, as adipose tissue undergoes hypertrophy, it becomes dysfunctional, altering the release of adipocyte-derived factors, known as adipokines. This dysfunction promotes low-grade chronic inflammation, exacerbates IR, and creates a hyperglycemic, proatherogenic, and prothrombotic environment. However, the fundamental cause of these phenomena remains unclear. This narrative review points to hypoxia as a critical trigger for the molecular changes associated with fat accumulation, particularly within visceral adipose tissue (VAT). The activation of hypoxia-inducible factor-1 (HIF-1), a transcription factor that regulates homeostatic responses to low oxygen levels, initiates a series of molecular events in VAT, leading to the aberrant release of adipokines, many of which are still unexplored, and potentially affecting peripheral insulin sensitivity. Recent discoveries have highlighted the role of hypoxia and miRNA-128 in regulating the insulin receptor in visceral adipocytes, contributing to their dysfunctional behavior, including impaired glucose uptake. Understanding the complex interplay between adipose tissue hypoxia, dysfunction, inflammation, and IR in obesity is essential for developing innovative, targeted therapeutic strategies.

## 1. Introduction

The discovery of insulin in 1921 revolutionized the treatment of diabetes mellitus [1]. However, shortly after its clinical application, it was observed that exogenous insulin did not uniformly lower glucose levels in all diabetic patients, leading to the introduction of the concept of insulin resistance (IR) to the scientific community [2,3]. Significant advances in understanding the mechanisms of insulin action occurred in the early 1970s with the identification of insulin receptors (INSR) on cell membranes, with their peculiar characteristic of receptors that contain intrinsic tyrosine kinase activity capable of intracellular signal transduction [4]. Despite extensive research into cellular insulin signaling, the precise molecular defect initiating IR remains incompletely understood, with both intracellular and extracellular factors implicated.

Obesity, characterized by chronic low-grade inflammation, is a predominant risk factor for IR, increasing the risk of chronic hyperglycemia, dyslipidemia, fatty liver, hypertension, cardiovascular disease, and certain types of cancers [5,6]. As obesity becomes an increasingly prevalent health issue, research has extensively focused on adipose tissue biology. Adipose tissue, beyond its role as a passive triglyceride storage site, actively participates in energy balance and systemic regulation of insulin sensitivity [7]. Studies have shown that in obesity, adipose tissue, especially visceral adipose tissue (VAT), contributes to whole-body glucose intolerance through dysfunctional adipocytes that exhibit impaired GLUT4-mediated glucose transport and insulin signaling. However, this impairment alone does not fully explain the pathogenesis of systemic IR in obese patients.

The discovery in 1987 of adipsin, an adipocyte-specific protein, and later leptin in 1994, marked a shift in understanding adipose tissue as a true endocrine organ [8]. Subsequent research identified a range of biomolecules released by adipocytes, collectively termed adipokines, which play roles in inflammation, systemic IR, and atherogenesis. The search for these biomolecules continues, with recent findings including not only hormones but also various effectors such as microRNAs, lipids, and metabolites produced by adipose cells, which influence local and systemic metabolic responses [9]. It has become clear that in obesity, adipose tissue becomes dysfunctional, leading to changes in gene and protein expression that result in a metabolically disadvantageous profile. The initial trigger for these changes remains a critical question. While factors such as endoplasmic reticulum (ER) stress, fibrosis, and mitochondrial dysfunction have been proposed as early events in the pathogenesis of obesity-related IR [10], substantial evidence points to hypoxia as a significant and intriguing candidate. This narrative review aims to explore the molecular mechanisms linking hypoxia to obesity-related IR, unraveling its complex network and offering further insights into its implications for metabolic health. To conduct this review, we manually retrieved the relevant literature from major electronic databases, including PubMed, Google Scholar, and Web of Science. We used various combinations of keywords such as “hypoxia”, “obesity”, “insulin receptor”, “adipose tissue”, “adipokine”, “dysfunction”, “inflammation”, and “microRNA” to guide our search. We selected studies directly related to these themes, incorporating novel and pertinent findings from our prior original research.

## 2. Physiology of INSR and IGF1 Receptor (IGF1R), and Downstream Signaling Pathways

Throughout life, insulin and insulin-like growth factors (IGFs) play crucial roles in regulating growth and metabolism by binding to their respective receptors on the cell membrane [11]. Insulin’s primary actions include reducing gluconeogenesis and glycogenolysis in the liver and increasing glucose uptake in skeletal muscle and adipose tissue. Regarding lipid metabolism, insulin promotes lipid synthesis in the liver and adipocytes while inhibiting the release of fatty acids from adipose tissue. Specifically, in adipose tissue, insulin is a key regulator of adipocyte function, promoting the differentiation of preadipocytes into adipocytes (adipogenesis). In mature adipose cells, it enhances the accumulation of energy in the form of triglyceride stores by stimulating glucose transport and triglyceride synthesis (lipogenesis). Insulin also inhibits lipolysis and increases the uptake of free fatty acids (FFAs) from circulating lipoproteins [5].

Many of these insulin-mediated actions occur through the interaction of INSR with INSR substrates, IRS1 and IRS2. This interaction results in alterations in enzyme activities through changes in their phosphorylation states, which arise from a combination of protein kinase inhibition and phosphatase activation. The INSR and IGF1R are highly homologous heterodimers composed of two α and two β subunits, stabilized by disulfide bonds [12]. These receptors derive from single-chain proreceptors encoded by the *INSR* and *IGF1R* genes, respectively. The α subunits are entirely extracellular and contain ligand-binding sites, while the transmembrane β subunits house an intracellular tyrosine kinase domain essential for catalytic activity and signal transduction [13].

Alternative splicing of exon 11 in the INSR mRNA produces a shorter INSR isoform (isoform A) and a longer isoform (isoform B) [14]. Isoform A is predominantly expressed in neurons, less-differentiated cellular progenitors, and malignant cells, while isoform B is more prevalent in mature tissues, including skeletal muscle, liver, and fat. In these mature tissues, isoform B plays prominent roles in glucose, lipid, and protein metabolism. While both INSR isoforms have similar insulin affinities, isoform A has a higher affinity for IGFs compared to isoform B. Despite their high degree of homology and many shared downstream signaling pathways, activation of each receptor results in distinct physiological outcomes. INSR is primarily involved in metabolic functions, whereas IGF1R is more involved in the regulation of mitogenesis and growth [11].

**Figure 1:**
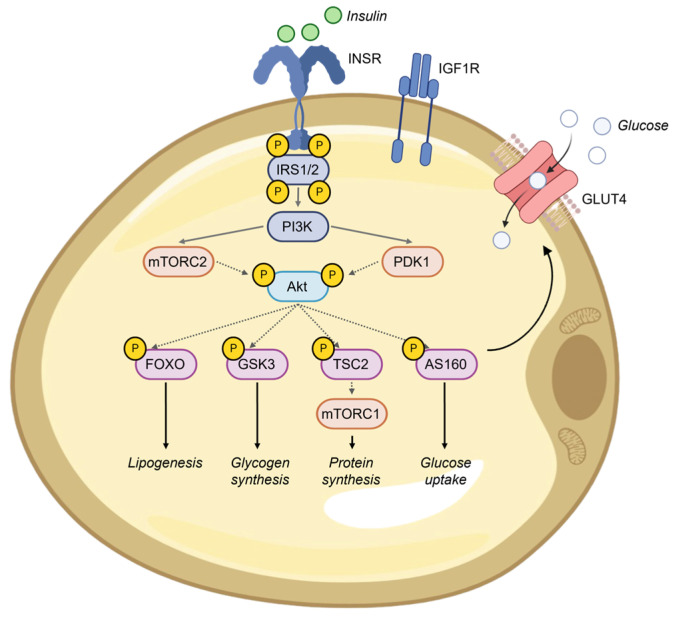
The key steps and effects of physiological insulin signaling in sensitive adipocytes. INSR, insulin receptor; IGF1R, IFG1 receptor; IRS1/2, insulin receptor substrates 1 and 2; PDK1, 3-phosphoinositide-dependent protein kinase 1; mTORC2 mammalian target of rapamycin complex 2; TSC2, tuberous sclerosis 2; GSK3, glycogen synthase kinase-3; AS160, Akt substrate of 160 kDa; mTORC1 mammalian target of rapamycin complex 1; GLUT4, glucose transporter 4.

Binding of insulin or IGFs to the extracellular domains of INSR, IGF1R, or INSR/IGF1R hybrid dimers induces conformational changes that lead to the rearrangement and activation of the intracellular β-subunits. This activation results in the phosphorylation of multiple tyrosine residues within the receptors and their immediate substrates, IRS1 and IRS2. A critical step in linking INSR activation to the downstream metabolic effects of insulin is the binding of phosphoinositide 3–kinase (PI3K) to tyrosine–phosphorylated IRS proteins, leading to the production of phosphatidylinositol (3,4,5)–triphosphate (PIP3). The downstream effects of PIP3 include the activation of 3-phosphoinositide-dependent protein kinase 1 (PDK1) and subsequent activation of Akt and other kinases, such as the mammalian target of rapamycin complex 2 (mTORC2) [15]. Full activation of Akt, which is necessary for the complete metabolic effects of insulin, requires PDK1-dependent phosphorylation at Thr308 and mTORC2-dependent phosphorylation at Ser473 [16]. For instance, Akt-induced phosphorylation inactivates glycogen synthase kinase-3 (GSK3) α/β, allowing the activation of glycogen synthase and increasing glycogen synthesis. Phosphorylation of forkhead box (FOX) O transcription factors results in their exclusion from the nucleus, preventing the expression of gluconeogenic genes in the liver [17] and regulating lipid catabolism and differentiation of adipocytes in adipose tissue [18]. Phosphorylation of tuberous sclerosis 2 (TSC2) activates mTORC1, which stimulates protein synthesis and suppresses autophagy. Additionally, phosphorylation of the Akt substrate of 160 kDa (AS160) facilitates the translocation of glucose transporter 4 (GLUT4) vesicles to the plasma membranes of skeletal muscle cells and adipocytes, enhancing glucose uptake [19]. Maintaining the structural and functional integrity of INSR signaling (Figure 1) is crucial for preserving insulin sensitivity and overall glucose homeostasis [20]. However, these aspects are disrupted in obesity.

## 3. Obesity and Obesity-Related Diseases: Pathophysiology and Molecular Determinants

Obesity is a chronic disease characterized by an excessive accumulation of body fat, typically defined by a body mass index (BMI) of 30 kg/m^2^ or higher [21]. It is linked to a wide range of prevalent illnesses, including type 2 diabetes mellitus (T2DM), hypertension, dyslipidemia, fatty liver disease, and cardiovascular disease, all of which share IR as a common underlying mechanism [6]. Additionally, obesity increases the risk of polycystic ovary syndrome (PCOS), which can lead to female infertility and pregnancy complications, including gestational diabetes [22]. Obesity is also associated with certain types of cancer and an increased susceptibility to severe infections, such as those related to COVID-19 [23]. Studies have demonstrated a significant association between obesity and vitamin D deficiency. While both obese and lean individuals may have comparable absolute levels of vitamin D, the concentration is typically higher in lean individuals due to less dilution and decreased storage of vitamin D precursors in adipose tissue. Furthermore, the conversion of these precursors into active vitamin D metabolites is often impaired in individuals with obesity [24]. Vitamin D deficiency may also promote adipogenesis, leading to increased body fat, which negatively impacts the clinical course and recovery from COVID-19, due to its immunomodulatory properties [25]. Currently, an estimated one-third of the global population is overweight or obese. Over the past 30 years, the prevalence of chronic illnesses due to obesity has exceeded that of nutritional deficiencies. Data analysis of 222 million patients by the School of Public Health at the Imperial College of London has recently estimated the global prevalence of underweight and obesity, noting significant increases in worldwide obesity prevalence since 1990, even in countries traditionally more affected by nutritional deficiencies. This shift highlights a global trend, with increasing obesity prevalence and declining underweight rates, even in low-income populations where malnutrition was previously dominated by undernutrition, such as in sub-Saharan Africa. The rise in obesity at younger ages underscores the need for effective prevention and management strategies to mitigate long-term exposure to obesity-related health risks in school-aged children and adolescents. Therefore, understanding the physiopathological mechanisms underlying diseases related to obesity, such as IR, is essential [26].

The World Health Organization (WHO) has identified obesity as a critical global research priority and one of the most pressing public health challenges of the coming decades [21]. Despite the prevalence and severe health consequences of obesity, our understanding of the associated diseases remains incomplete, particularly concerning the primary causes of IR in obese individuals [10]. As discussed in Barbara Kahn’s Banting Lecture [5], a significant link between obesity and related diseases is the dysfunction of VAT, which expands and becomes enlarged, inflamed, and metabolically impaired. Under normal physiological conditions, adipocytes, the primary cells in VAT, are highly responsive to insulin, supporting the storage of energy as triglycerides by stimulating glucose transport and triglyceride synthesis. In obesity, however, IR leads to reduced insulin-stimulated glucose transport in adipocytes. This defect is coupled with reduced glucose uptake in skeletal muscle and impaired suppression of gluconeogenesis and glycogenolysis in hepatocytes [5]. Generally, these metabolic alterations result from impaired INSR signaling in insulin-target tissues. However, in skeletal muscle, IR often occurs without changes in INSR protein expression, instead marked by defective insulin-signaling and impaired insulin-stimulated translocation of GLUT4 to the plasma membrane [5,9]. In VAT adipocytes from obese individuals and animal models, significant downregulation of INSR and GLUT4 expression has been observed, directly contributing to IR and glucose intolerance [5,9,27]. Furthermore, impaired insulin function and glucose uptake in VAT adipocytes can lead to abnormal secretion of adipose tissue-derived products, including cytokines and adipokines, which adversely affect glucose homeostasis and insulin sensitivity at both local and systemic levels. These endocrine products, detailed in the subsequent sections of the manuscript, collectively play significant roles in regulating energy balance, reproductive function, tumorigenesis, vascular tone, immune functions, and hemostasis [28].

## 4. The Role of Hypoxia in Inflaming VAT

The increasing prevalence of obesity and its associated comorbidities, particularly T2DM, has spurred significant interest in the endocrine functions of VAT, which was previously considered merely a passive lipid storage site. Currently, over 600 adipokines and cytokines have been identified through proteomic studies. Ongoing research is exploring additional VAT-derived products, such as fatty acids, intermediate metabolites, and microRNAs (miRNAs), which may play crucial roles in inter-organ metabolic communications [29,30]. In obesity, the altered secretion of adipokines and cytokines from dysfunctional VAT supports a chronic inflammatory, pro-thrombotic, and pro-tumorigenic state. This contributes to other pathophysiological processes typical of IR-related conditions, including hyperglycemia, dyslipidemia, and hypertension [29].

Oxygen (O_2_) is essential for most eukaryotic organisms to maintain normal cellular activities. A limited oxygen supply or an increased rate of oxygen consumption leads to hypoxia. Hypoxia typically refers to a relatively low oxygen concentration (commonly < 2% O_2_) compared to the normal level at which a given organ, tissue, or cell physiologically functions (not equivalent to the ambient level of 21% O_2_) [31]. Although adaptive mechanisms may facilitate survival under these conditions, growing evidence implicates hypoxia as a key factor in the pathogenesis of major causes of morbility and mortality, including cancer, myocardial infarction, chronic heart and kidney diseases, as well as metabolic diseases [32,33]. Indeed, a key mechanism implicated in VAT dysfunction and abnormal release of biologically active adipose-derived products is hypoxia. As VAT expands in obesity, the distance between adipocytes and the vasculature increases, reducing oxygen tension and leading to hypoxia. Oxygen deprivation below physiological levels contributes to the aberrant secretion of pro-inflammatory cytokines and adipokines by hypertrophic adipocytes [34]. Hypertrophic adipocytes can grow to diameters of 150–200 μm, exceeding the typical oxygen diffusion limit of 100–200 μm. Landmark studies using validated genetic obesity models, in which oxygen levels were directly measured in visceral fat using a needle-type oxygen microsensor, demonstrated a significant difference in interstitial oxygen levels. In obese mice, epididymal fat depots exhibited oxygen levels of 15.2 mmHg (~2% O_2_), compared to 47.9 mmHg (~7% O_2_) in their lean counterparts. This represents a 70% reduction in oxygen tension within the visceral fat of obese mice, which is attributed not to systemic hypoxia, but rather to inadequate local blood flow and vascular support [35]. Corresponding human studies, although focused on oxygen levels in abdominal subcutaneous fat, have similarly documented lower oxygen levels in obese individuals’ adipose tissue. This reduction correlates with a decrease in capillary density, paralleling findings in obese mice [36]. This hypoxic environment induces oxidative stress, ER stress, and the activation of the unfolded protein response (UPR), collectively contributing to cell dysfunction. The ER is essential for the proper folding, maturation, and assembly of proteins, as well as for maintaining cellular calcium homeostasis. Hypoxia can alter these processes by causing protein misfolding, as the formation of disulfide bonds, which require oxygen, becomes compromised. This leads to ER stress and triggers the UPR [37]. This cascade of events aims to manage the accumulation of unfolded and misfolded proteins in the ER lumen, thereby alleviating cellular stress and restoring homeostasis. A key aspect of the UPR involves the activation of mTORC1, a critical regulator of protein synthesis and cell growth that functions downstream of the PI3K/Akt signaling pathway (Figure 1). Additionally, ER stress can reciprocally influence mTORC1 and the PI3K/Akt pathway, resulting in suppressed insulin signaling and reduced Akt phosphorylation [38]. While the UPR initially serves a protective role, prolonged ER stress can lead to cell death [39]. Hypoxia also targets mitochondria, where it induces overproduction of reactive oxygen species and calcium overload, impairing ATP generation through oxidative phosphorylation and resulting in an energy deficit [40]. Moreover, a significant cellular response to hypoxia includes alterations in chromatin-remodeling proteins, leading to widespread changes in gene expression [34]. In this context, hypoxia-inducible factor-1 (HIF-1) is a central nuclear transcription factor mediating cellular responses to hypoxia, including the upregulation of genes relevant to obesity and diabetes [41,42]. HIF-1 is composed of two subunits: HIF-1β, which is constitutively expressed, and HIF-1α, which is rapidly degraded under normal oxygen conditions but stabilized in response to low oxygen levels, forming the functional transcription factor. In addition to HIF-1, other transcription factors, including the nuclear factor kappa-light-chain-enhancer of activated B cells (NF-κB), cAMP response element-binding protein (CREB), and their chromatin partner high-mobility group A1 (HMGA1), are involved in the hypoxic response. These factors often engage in cross-talk, particularly in pathways related to inflammation and immunity [42].

Approximately seventy genes have been identified as hypoxia-sensitive via the hypoxia response elements (HREs) of HIF-1, including those encoding proteins involved in angiogenesis, cell proliferation and survival, apoptosis, vascular tone, as well as glucose and energy metabolism [43]. Specific examples of hypoxia-sensitive genes include GLUT1, which facilitates basal glucose uptake independent of insulin [44], along with leptin [45], adiponectin [35,46], the major angiogenic factor vascular endothelial growth factor (VEGF) [47], and several enzymes involved in glucose metabolism such as phosphofructokinase [48]. Elevated levels of HIF-1α have been observed in the adipose tissue of obese mice and humans, correlating with increased adiposity and indicating the progressive worsening of hypoxia with the severity of obesity [9]. However, HIF-1’s role in obesity extends beyond metabolic regulation. Recent research suggests that HIF-1α activation in low oxygen environments may promote the production of amyloid-beta (Aβ) and tau hyperphosphorylation, markers of Alzheimer’s Disease (AD), which have also been found in the brains of diabetic individuals [49]. This points to a potential pathological pathway linking AD and T2DM through HIF-1.

To exert its transcriptional activities, HIF-1 can bind directly to DNA or associate with the chromatin factor HMGA1 [42]. Studies in murine 3T3-L1 adipocytes and human embryonic kidney HEK-293 cells have shown that the gene expression of Visfatin and VEGF, induced by HIF-1α activation, increases only when HMGA1 is overexpressed. This suggests a synergistic role of HIF-1α and HMGA1 in the hypoxic response [42]. Hypoxia can also activate the transcription factor NF-κB [50], which consists of two Rel family proteins, p65 and p50, typically held inactive in the cytoplasm by the inhibitor IκBα. Under hypoxic conditions, NF-κB is released from IκBα, leading to nuclear translocation and the transcription of genes involved in the inflammatory cascade, including tumor necrosis factor α (TNF-α), and interleukine (IL)-1, and IL-6. These cytokines can activate serine kinases such as the IκB kinase (IKK), Jun N-terminal kinase (JNK), protein kinase C (PKC), and 70-kDa ribosomal protein S6 kinase (p70S6K), which inhibit IRS1 function and exacerbate IR [51]. Additionally, NF-κB inhibits peroxisome proliferator-activated receptor γ (PPARγ), a nuclear receptor crucial for adipocyte differentiation and insulin sensitivity, thereby suppressing the transcription of genes involved in glucose and lipid metabolism [51,52]. It is worth noting that prolonged hyperglycemia can activate the NF-κB pathway, contributing to both microvascular and macrovascular complications in T2DM in a vicious circle [53,54].

Obesity-related complications involve distinct molecular mechanisms, all sharing a common basis of low-grade chronic inflammation linked to adipose tissue dysfunction. Adipocyte hypoxia activates HIF-1 and NF-κB, stimulating the release of pro-inflammatory signals that alter the stromal vascular environment. Physiologically, adipose tissue hosts a dynamic composition of resident cells, including various immune cells such as T helper cells (CD4+ T cells), cytotoxic T cells (CD8+ T cells), B regulatory cells (B reg cells), T regulatory cells (T reg cells), and M2 macrophages. These cells maintain adipose tissue homeostasis by orchestrating the secretion of cytokines and adipokines to preserve an anti-inflammatory environment [55]. In obesity, the metabolic and endocrine functions of adipose tissue shift towards a pro-inflammatory and pro-fibrotic state. Hypoxia-induced stress or death of VAT adipocytes triggers macrophage infiltration and activation, forming crown-like structures around apoptotic adipocytes. This macrophagic infiltration is characterized by both increased recruitment and decreased egress of macrophages, mediated by chemokines like the monocyte chemoattractant protein-1 (MCP-1), which attracts macrophages, and the macrophage migration inhibitory factor (MIF), which prevents their departure [56]. Another hallmark of immune alterations in obesity is the transition of macrophages from an M2 to an M1 phenotype, further remodeling the adipose tissue microenvironment and contributing to the complex pro-inflammatory network [57,58]. Activation of HIF-1α is implicated in the shift of M2 macrophages towards the pro-inflammatory M1 phenotype. M2 macrophages release anti-inflammatory cytokines such as IL-1 receptor antagonist, IL-4, IL-10, and TGF-β, and activate arginase 1 (Arg1) to inhibit inducible nitric oxide synthase (iNOS) activity. In contrast, M1 macrophages secrete pro-inflammatory cytokines such as IL-1β, IL-6, IL-12, TNF-α, and MCP-1, along with reactive oxygen species generated by iNOS [59]. This increases the risk of cardiovascular diseases and atherosclerosis [60]. Additionally, hypoxia, mediated by HIF-1, alongside insulin signaling through Akt and inflammatory cytokines, also influences the expression and/or activity of endothelial nitric oxide synthase (eNOS), a key regulator of endothelial and vascular homeostasis [61]. M1 macrophages also release VEGF, which is associated with angiogenic and tumorigenic processes [62]. This inflammatory milieu contributes to the link between obesity and cancer, now the leading cause of death in obese patients, surpassing cardiovascular disease mortality rates [63]. Other immune cells also contribute to local inflammation. Neutrophils, abundant in circulation, migrate to specific sites and exacerbate chronic inflammation by promoting macrophage recruitment and secreting proteases like elastase, myeloperoxidase, and calprotectin. Neutrophils from obese individuals show increased superoxide secretion compared to their lean counterparts, promoting cell apoptosis and macrophage activation, thus amplifying the pro-inflammatory response [64]. Regarding lymphocytes, regulatory T cells (Treg cells) are abundant in the adipose tissue of lean/normal mice compared to their presence in the spleen and lymph nodes [65]. However, in insulin-resistant genetic models of obesity, such as the *ob/ob* and *db/db* mice, and in high-fat diet-induced obese mice, Treg cells in adipose tissue are markedly diminished [65]. Characterized by specific cell markers (CD4+ CD25+) and the key lineage-defining transcription factor Foxp3, Treg cells suppress the activation and proliferation of effector T cells, maintaining tolerance to self-antigens [66]. In contrast, individuals with severe obesity and T2DM exhibit a significant reduction in circulating mucosal-associated invariant T (MAIT) cells, which show an activated pro-inflammatory phenotype, indicating aberrant MAIT cell activation in obesity and T2DM [67]. In obese patients, MAIT cells are more abundant in adipose tissue than in the blood and express an IL-17-dominant immune profile. Bariatric surgery in obese patients not only improves metabolic parameters but also increases circulating MAIT cell frequency while reducing cytokine production [67]. Collectively, these immune alterations link obesity with autoimmune or inflammatory diseases, such as multiple sclerosis, Crohn’s disease, and ulcerative colitis, where IL-17 and related signaling are functionally implicated [68,69].

## 5. Hypoxia-Induced Adipokine Dysregulation and IR

Adipokines, biologically active molecules produced by adipose tissue cells, play a crucial role in regulating immune and vascular functions as well as numerous metabolic processes. These molecules are central to the continuous growth, regression, and remodeling of blood vessels, acting as angiogenic growth factors [70]. In white adipose tissue (WAT), angiogenic adipokines facilitate energy storage and increase vascular density [71]. Conversely, in brown adipose tissue (BAT), this angiogenic response aids in energy expenditure [72]. Additionally, the blood vessels within adipose tissue provide a supportive environment for the differentiation of adipocyte progenitors into pre-adipocytes and adipocytes [73]. However, the compensatory angiogenesis in response to tissue expansion in obesity can only sustain adequate oxygenation to a certain extent. Beyond this limit, local hypoxia, characterized by reduced blood flow, decreased capillary density, and increased vasoconstriction (often mediated by angiotensin II), takes place [34,70]. This chronic low oxygen state stabilizes HIF-1α, which subsequently upregulates pro-inflammatory and pro-angiogenic factors via HREs [74]. Notable examples of factors under the control of HREs include leptin, adiponectin, VEGF-A, IL-6, apelin, visfatin, and resistin. These factors contribute to chronic inflammation and macrophage infiltration into adipose tissue [34]. Concurrently, the levels of anti-inflammatory adipokines decrease, setting the stage for adipocyte death [75]. Once secreted into the extracellular space, adipokines and cytokines, deregulated by hypoxic stimuli, interact with specific receptors on recipient cells, triggering a series of intracellular pathways that ultimately impair insulin signaling. Key pathways implicated include the activation of IKK/NF-κB and protein kinases JNK, PKC and mitogen-activated protein kinases (MAPK), which can phosphorylate serine residues and inhibit INSR and IRS1, key components in insulin signaling [10]. Additionally, Janus kinase/signal transducers and activators of transcription JAK1/JAK2/STAT1 and STAT3, activated by cytokines like INFγ and IL-6, disrupt the interaction of INSR with IRS proteins by repressing tyrosine kinase activity and promoting IRS degradation [10,76]. To date, more than 600 secreted cytokines and adipokines potentially influenced by hypoxia have been identified, highlighting the complexity of adipose regulatory networks. However, for most of these biomolecules, there is no clear explanation for their biological effect, and their circulating patterns in obese patients are still being elucidated. Very recently, a role for prothymosin-α (Prot-α), an innate “alarmin” over-released by hypoxic adipocytes [22], has been proposed in the development of obesity-related IR and inflammation. Formerly identified as a multifunctional protein, Prot-α enhances T-helper type 1 (Th1) adaptive immune responses and stimulates the differentiation of monocytes into dendritic cells, promoting the production and secretion of pro-inflammatory cytokines [77]. Elevated levels of circulating Prot-α in obese patients, even before clinical manifestations of IR or changes in conventional metabolic indices and circulating cytokines, suggest its potential as a sensitive biomarker for early detection and monitoring of obesity-related inflammation and IR [78]. Table 1 summarizes the effect of hypoxia and obesity on some of the most studied adipokines, with proposed roles in IR and inflammation.

## 6. Dysregulation of MiRNAs Targeting INSR in Obesity

In addition to cytokines, miRNAs play a significant role in the pathogenesis of IR and various physiological and pathological processes, such as development, aging, death, and inflammation. MiRNAs are evolutionarily conserved 19–25 nt noncoding RNAs that regulate gene expression and translation [91]. Typically, miRNAs negatively influence gene expression by binding to specific sites within the 3′ untranslated region (3′ UTR) of messenger RNAs (mRNAs), leading to the destabilization, translational repression, or cleavage of the target mRNA [92]. However, there are instances where miRNAs upregulate gene expression [93]. Emerging evidence suggests that miRNAs, released by adipose cells in exosomes and other vesicles, play crucial roles in maintaining glucose homeostasis and influencing macrophage polarization in a paracrine manner [94,95]. The precise mechanisms of action have been elucidated for only a subset of these miRNAs, and discrepancies in study results may be due to the clinical characteristics of the populations studied or the method used for normalizing miRNA expression [96]. Significant miRNAs in obesity-related IR and inflammation include miR-29a, miR-330-5p, and miR-495, all of which are upregulated in macrophage-derived exosomes from obese mice [97,98,99]. MiR-330-5p downregulates the TIM-3 gene, promoting a shift from the anti-inflammatory M2 macrophage phenotype to the pro-inflammatory M1 phenotype, exacerbating IR [98]. Similarly, MiR-495 promotes M1 macrophage polarization by targeting the *FTO* gene, whose polymorphisms are associated with comorbidities in obese patients [99,100]. MiR-29a affects cellular insulin sensitivity by targeting peroxisome proliferator-activated receptor delta (PPARδ) [97]. Moreover, miR-27a and miR-130b regulate macrophage polarization and adipose tissue inflammation [101,102]. MiR-27a, suppresses PPARγ expression, promoting M1 polarization and increasing the expression of NF-κB and Toll-like receptor 4 (TLR4), both typically inhibited by PPARγ activation [101]. NF-κB and TLR4 are inflammatory mediators that significantly contribute to obesity-induced IR by increasing the secretion of pro-inflammatory cytokines [103]. Other studies also support the involvement of the PPAR-γ pathway [104]. MiR-130b’s increased expression in macrophages from high-fat diet-fed mice correlates with enhanced M1 polarization and decreased M2 polarization, with 3′ UTR PPARγ mRNA identified as a direct target of miR-130b [102]. Inhibition of miR-130b attenuates M1 polarization and enhances M2 polarization in high-fat diet-fed mice [102].

**Figure 2:**
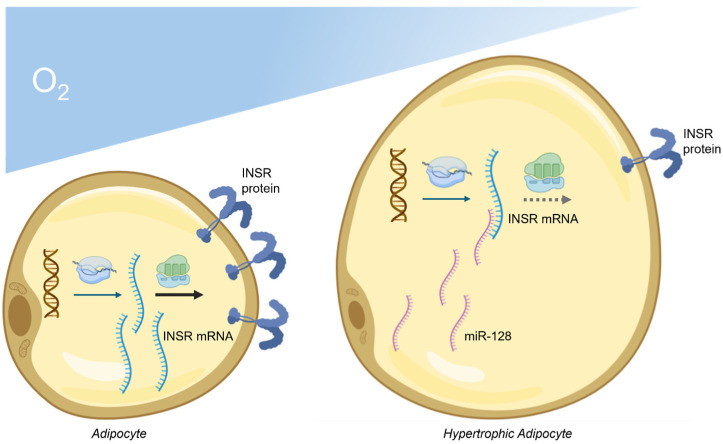
Morphological and functional changes occurring in mature visceral adipocytes following transition into a hypertrophic state, as commonly observed in obesity. This transition involves an increase in cell size and a decrease in oxygen (O_2_) tension within the cells, ultimately leading to a hypoxic environment. In hypoxic adipocytes, overexpression of miR-128 promotes the degradation of INSR mRNA, leading to reduced production of INSR protein and subsequent development of IR.

These findings highlight the multifaceted roles of miRNAs in the pathogenesis of IR in obesity, often involving the modulation of inflammatory pathways. Deregulated circulating miRNAs offer potential as disease biomarkers, and ongoing research is delving into their intracellular mechanisms, with evidence suggesting that hypoxia is a primary driver of their deregulation. The first report of an miRNA with physiological relevance in glucose homeostasis and insulin signaling regulation in adipose tissue was published in 2006, focusing on miR-278 in *Drosophila melanogaster* adipocytes. Teleman et al. demonstrated that miR-278 targets two sites on the 3′ UTR of *expanded*, an essential gene whose membrane-associated product affects insulin signal transduction and energy metabolism [105]. Mutant flies lacking miR-278 overexpressed *expanded* in adipose tissue and exhibited abnormally high levels of circulating insulin and trehalose (a sugar molecule), mimicking mammalian IR [105]. Subsequent studies in mammals confirmed miRNAs’ involvement in post-transcriptional regulation of insulin signaling components, including the INSR, in adipocytes and other insulin-responsive cells. For instance, miR-27b was significantly upregulated in an insulin-resistant model of murine 3T3-L1 adipocytes and VAT tissue of high-fat diet-fed obese mice [106]. Bioinformatic prediction analysis, validated by a luciferase reporter gene assay, showed that miR-27b directly targets the 3′ UTR of the INSR mRNA, inducing premature decay of this transcript. More recently, miR-128 has been identified as a key intracellular miRNA in visceral adipocytes upregulated by hypoxia [9]. By binding to the 3′ UTR of INSR mRNA, miR-128 markedly accelerates its decay in hypoxic visceral adipocytes, reducing INSR levels and impairing insulin signaling (Figure 2). Obese humans and mice express higher levels of miR-128 in VAT compared to their lean counterparts, along with reduced INSR expression and GLUT4 mediated glucose uptake. Hypoxia-induced miR-128 upregulation is proposed as a mechanism of hyperglycemia and glucose intolerance in obesity, suggesting that blocking miR-128 in VAT may mitigate obesity-induced IR [9]. Other miRNAs, such as miR-15b [107], miR-195 [108] and miR-96 [109], also target INSR transcripts and contribute to IR in human hepatocytes pre-treated with saturated fatty acids and liver tissues of obese mice on a high-fat diet. Collectively, these miRNAs form a complex network regulating insulin sensitivity and inflammation in obesity. Understanding their precise roles and mechanisms offers potential avenues for therapeutic interventions targeting obesity and its related metabolic disorders.

In addition to miR-128, other miRNAs that function intracellularly or extracellularly are also influenced by hypoxia and have been linked to inflammation and/or IR in obesity. These miRNAs are detailed in Table 2.

## 7. Clinical Implications and Future Directions

Quantifying circulating adipokines and cytokines offers significant potential for the clinical management of obese patients, offering substantial advantages over traditional laboratory tests for assessing cardiometabolic risk [78]. These biomarkers provide valuable insights into a patient’s dynamic inflammatory and metabolic status, which can evolve throughout their lifetime due to lifestyle changes or the onset of new chronic illnesses. Recent studies have identified a relationship between levels of adipokines and cytokines—markers of IR and chronic low-grade inflammation—and the degree of visceral adiposity, as indicated by BMI [9,115]. Additionally, abnormal release patterns of these biomarkers can be partly normalized following weight loss interventions [115]. Weight loss reduces adipocyte size, potentially alleviating the hypoxic conditions commonly observed in obese VAT [116]. On a molecular level, early hypoxia-induced alterations can be reversed by restoring normal oxygen levels, which involves suppressing miR-128 and reestablishing normal INSR expression and insulin signaling in visceral adipocytes [9]. In addition to the reoxygenation that follows weight loss, other strategies could also address metabolic dysfunctions in VAT. For instance, using inhibitors for miRNAs that are overexpressed in obese adipocytes (e.g., antagomiRs targeting miR-128 [9]) or mimics for those that are underexpressed (e.g., mimics for miR-182-5p/miR-30c-2-3p [111]) may be effective. Direct targeting of adipokines and cytokines through pharmaceuticals and dietary interventions could also be beneficial. Moreover, it is conceivable that some cardiometabolic health benefits of the Mediterranean diet, even without significant calorie reduction and associated weight loss or VAT reoxygenation, may derive from specific dietary components and phytochemicals that directly influence oxygen sensing, oxidative stress, insulin signaling, or inflammation [6]. Investigating the molecular mechanisms underlying IR and inflammation in obesity through hypoxia-related pathways could reveal new treatment opportunities for obesity and its related complications.

## 8. Limitations

This narrative review intentionally incorporates a degree of subjectivity in selecting and interpreting studies. It does not aim to exhaustively cover all molecular alterations linked to the pathogenesis of IR, or the advancements in understanding insulin signaling components downstream of the INSR, which may be altered in obesity. Similarly, it does not explore the implications of ER stress and mitochondrial dysfunction, potentially influenced by hypoxia. These topics are covered in other reviews. Additionally, the generalizability of our findings to obese populations with diverse genetic or ethnic backgrounds, varying comorbid conditions, or different laboratory settings—especially concerning the equipment used and the criteria for defining hypoxic and normoxic states in adipocyte studies—remains uncertain.

## 9. Conclusions

**Figure 3:**
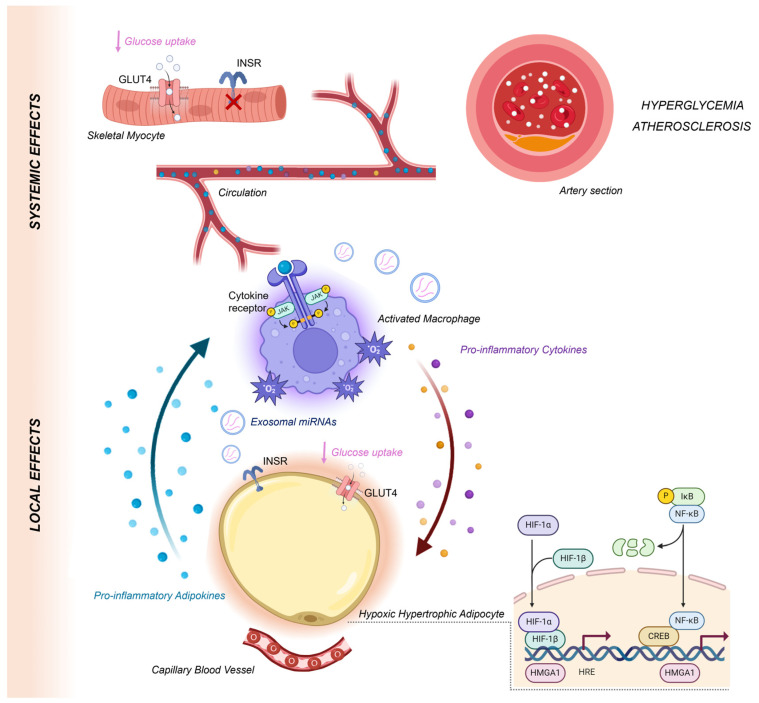
The interplay between hypoxia, inflammation, and metabolic dysregulation at the core of the pathogenesis of obesity-related IR. As obesity progresses and fat mass increases, visceral adipocytes enlarge and become increasingly distant from capillary blood vessels, leading to hypoxia and subsequent cellular dysfunction. Hypoxic adipocytes exhibit reduced expression and activity of the INSR, which impairs the translocation of the insulin-responsive glucose transporter GLUT4 to the plasma membrane, reducing glucose uptake. Dysfunctional adipocytes also release an abnormal pattern of pro-inflammatory adipokines and cytokines into the extracellular milieu and the circulatory system. The aberrant biosynthesis and secretion of these pro-inflammatory biomolecules are modulated by HIF-1, which is overactivated in hypoxia and partners with other nuclear factors, such as NF-κB, CREB, and HMGA1, to bind to HREs. This dysregulation in adipokine secretion disrupts systemic insulin signaling, particularly affecting skeletal muscle, another primary site for glucose uptake alongside adipose tissue, thereby further impairing whole-body glucose homeostasis. Additionally, the dysfunctional behavior of hypoxic adipocytes promotes the infiltration and activation of macrophages within visceral adipose tissue. These macrophages then secrete pro-inflammatory cytokines, reactive oxygen species, and exosomal miRNAs, intensifying both local and systemic inflammation. This pathological interplay between adipocytes and immune cells contributes to the development of IR, hyperglycemia, and the onset and progression of atherosclerosis in obese individuals.

The interplay between hypoxia, inflammation, and metabolic dysregulation is central to the pathogenesis of obesity-related IR (Figure 3). Adipose tissue, particularly VAT, acts as an active endocrine organ, secreting a wide range of bioactive molecules that influence systemic metabolic processes. Hypoxia, a common feature of expanding adipose tissue in obesity, triggers a cascade of molecular events that exacerbate inflammation and IR. The dysregulation of adipokines and miRNAs in response to hypoxia plays a crucial role in these processes. Adipokines such as leptin, adiponectin, and VEGF, along with pro-inflammatory cytokines, contribute to the inflammatory milieu and metabolic dysregulation observed in obesity. Additionally, miRNAs modulate gene expression, influencing INSR signaling and inflammatory pathways. Despite significant progress in understanding these mechanisms, many questions remain unanswered. The exact sequence of molecular events leading to IR, the primary factors involved, and the interplay between different signaling pathways still need to be elucidated. Future research should focus on integrating these findings to develop comprehensive models of obesity-related IR and identifying potential therapeutic targets.

## Figures and Tables

**Table 1:** Effect of hypoxia on the release of adipokines from adipose cells and their influence on insulin signaling and inflammatory responses in obesity.

	Hypoxia	Association with IR	Association with Insulin Sensitivity	Type of Inflammatory Response	Effect on Angiogenesis	Ref. No.
Leptin	↑	+	−	Pro-inflammatory	↑	[79,80]
Resistin	↑	+	−	Pro-inflammatory	↑	[81,82]
Apelin	↑	−	+	Anti-infiammatory	↑	[83]
Vaspin	↑	−	+	Anti-infiammatory	↑	[84]
Retinol binding protein 4	↑	+	−	Pro-inflammatory	↑	[85,86]
Adiponectin	↓	−	+	Anti-infiammatory	↑	[87]
Visfatin	↑	+	+	Pro-inflammatory	↑	[88]
Omentin	↓	−	+	Anti-infiammatory	↑	[89]
Nesfatin	↓	−	+	Anti-infiammatory	↑	[90]

**Table 2:** Effect of hypoxia on key miRNAs that may influence inflammation and insulin sensitivity.

MiRNA	Location	Hypoxia	Biological Effect	Ref. No.
miR-142-3p	intracellular	↓	Promotion of apoptosis, inflammation and fibrosis	[110]
miR-182-5p	intracellular	↓	Inhibition of adipocyte insulin signaling by interfering with PI3K/Akt	[111]
miR-30c-2-3p	intracellular	↓	Inhibition of adipocyte insulin signaling by interfering with PI3K/Akt	[112]
miR-210-3p	intracellular	↑	Promotion of adipose tissue inflammation by activating NF-κB	[112]
miR-27a/b	intracellular	↑	Inhibition of preadipocyte differentiation by suppressing PPARγ	[113]
miR-210/92a	exosomal	↓	Inhibition of adipose browning and energy expenditure	[114]
